# Molecular Mutations in Histiocytosis: A Comprehensive Survey of Genetic Alterations

**DOI:** 10.1007/s12033-024-01072-2

**Published:** 2024-02-20

**Authors:** Padmini Pai, Arnav Nirmal, Lian Mathias, Siya Jain, Manasa Gangadhar Shetty, Babitha Kampa Sundara

**Affiliations:** 1 Department of Biophysics, Manipal School of Life Sciences, Manipal Academy of Higher Education, Manipal, Karnataka 576104 India; 2 Manipal School of Life Sciences, Manipal Academy of Higher Education, Manipal, Karnataka 576104 India

**Keywords:** Histiocytosis, Myeloid, Lesions, Neoplasms, Point mutation, Pathogenesis

## Abstract

Histiocytosis represents a group of uncommon disorders characterized by the abnormal accumulation of specialized immune cells, such as macrophages, dendritic cells, or monocyte-derived cells, in various tissues and organs. Over 100 distinct subtypes have been documented, each displaying a broad spectrum of clinical presentations and histological characteristics. Till today, histiocytosis has been addressed through a combination of chemotherapy, radiotherapy, and surgery, with varying responses from individual patients. Due to its atypical symptoms, it has been prone to misdiagnosis. Advances in our understanding of the cellular and molecular aspects of these conditions are paving the way for improved diagnostic methods and targeted therapies. Researchers have extensively investigated various mutations in patient samples. However, no paper has yet provided a comprehensive summary of the collective analysis of mutations and pathways. Hence, this paper consolidates research efforts that specifically concentrate on gene mutations identified in patient samples of different subtypes of histiocytosis. These insights are essential for developing targeted therapies and improving diagnosis. Further, it provides potential insights to enhance the development of more effective therapeutic approaches for rare diseases.

**Graphical Abstract:**

Parts of the figure were drawn by using pictures from Servier Medical Art. Servier Medical Art by Servier is licensed under a Creative Commons Attribution 3.0 Unported License (https://creativecommons.org/licenses/by/3.0/).

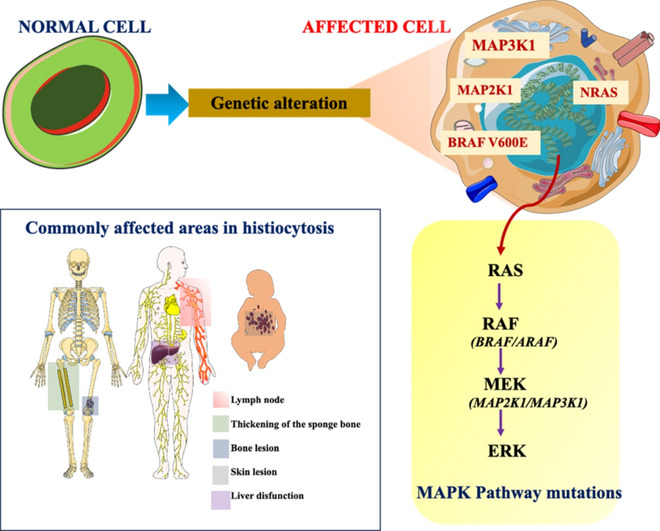

## Introduction

**Fig. 1:**
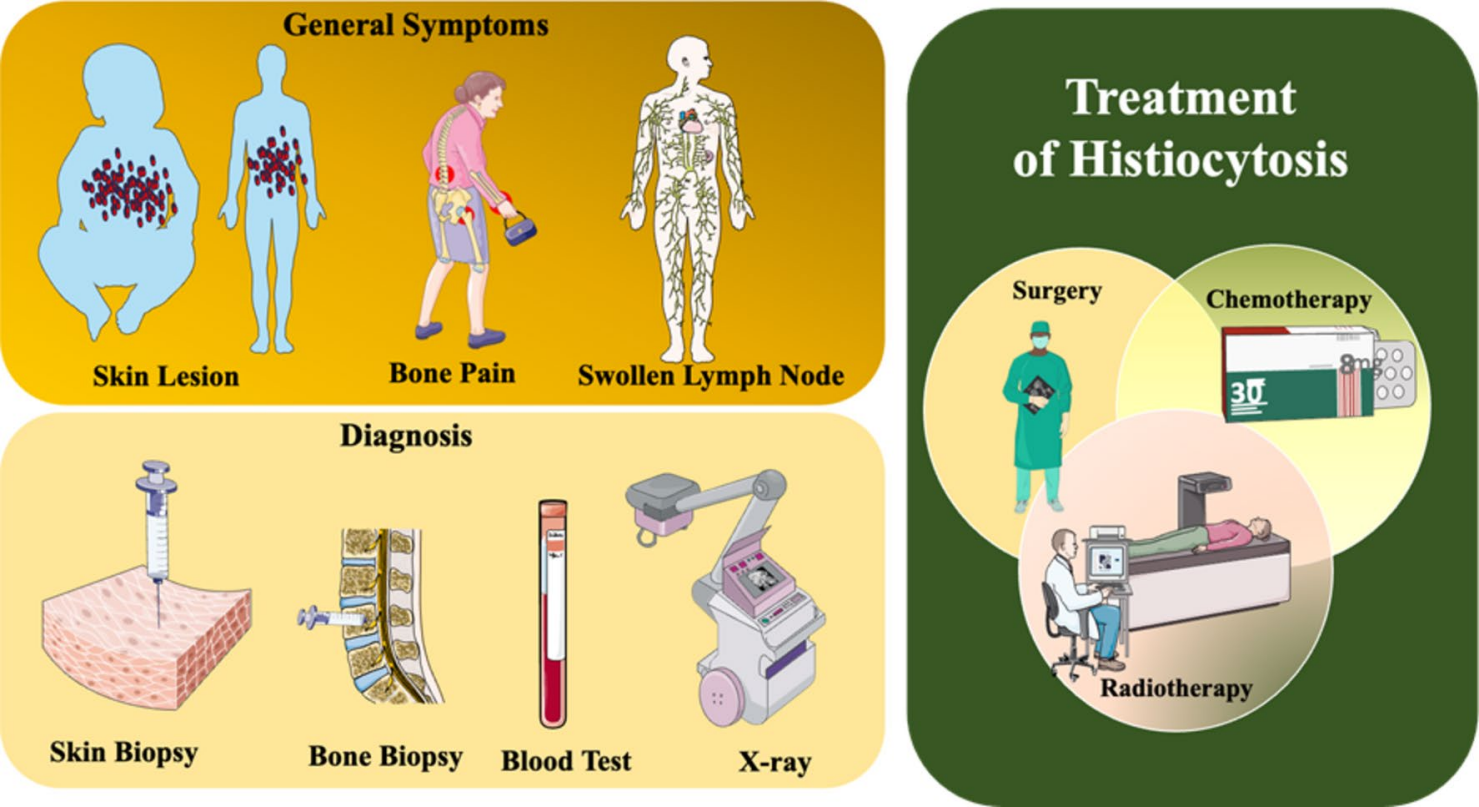
Treatments, diagnosis and general symptoms of histiocytosis. Parts of the figure were drawn by using pictures from Servier Medical Art. Servier Medical Art by Servier is licensed under a Creative Commons Attribution 3.0 Unported License (https://creativecommons.org/licenses/by/3.0/)

Histiocytosis is a varied group of rare disorders marked by myeloid cell overgrowth and infiltration of nearly every organ of the human body. It may start at any age affecting both adults and children. It can also be explained as the proliferation of histiocytes (immune cells derived from hematopoietic processes) leading to the formation of tumors. These cells are capable of infiltrating any tissue or organ but have a special liking towards the central nervous system (CNS), skin, heart, lymph nodes, and bone [1]. The initial publication by the working group of the Histiocyte Society, in 1987, classified histiocytosis in three categories: langerhans cell histiocytosis (LCH), non-langerhans cell histiocytosis (non-LCH) and malignant histiocytosis (MH) [2]. However, in light of recent advancements, the classification is based on clinical, pathological, histological, radiographic, genetic, phenotypic, and molecular alterations. The histiocytic disorders are now classified under five groups [3]. LCH and erdheim-chester disease (ECD) consists of the “L group”; the cutaneous and mucocutaneous or the “C group” includes the juvenile xanthogranuloma (JXG) family; the MH or the “M group”, the Rosai-Dorfman-Disease (RDD) or the “R group”, lastly is the group of hemophagocytic lymphohistiocytosis (HLH) and macrophage activation syndrome or the “H group” [4]. The intrusion of the mutated immune cells is characterized by a rash on the scalp, lesions on the organ or tissue affected, discharge from the ear, loss of appetite, and fever. In children sometimes the pituitary gland of the brain might be affected, leading to diabetes insipidus in young children. Certain other symptoms might include weight loss, headache, slow growth, mental deterioration, seizures, jaundice and vomiting. Figure 1 illustrates the treatments, common symptoms and diagnosis, associated with histiocytosis.

**Fig. 2:**
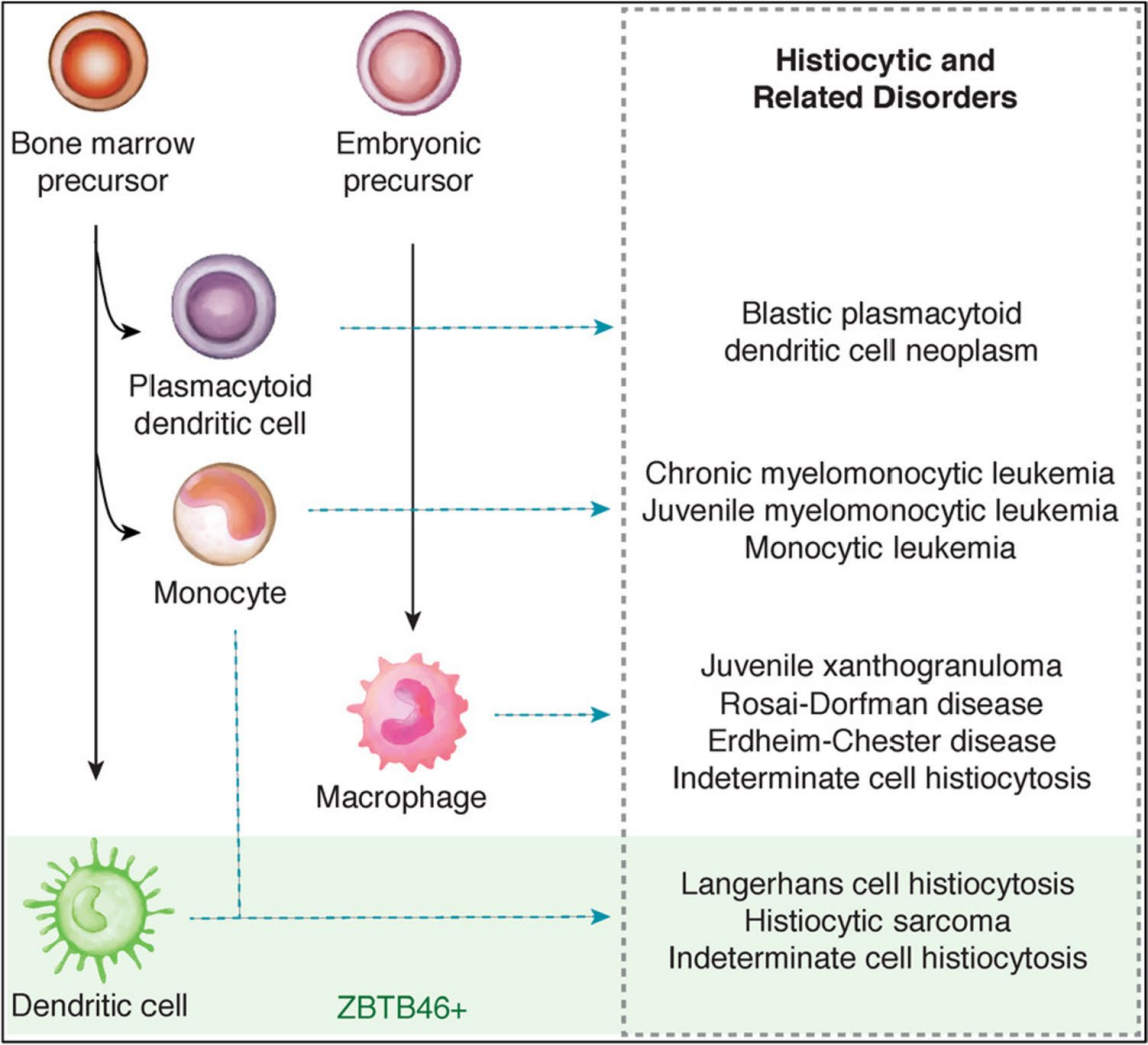
Proposed diagram illustrating the cell identity within histiocytic disorders (Adapted with permission from [9])

Genetic alterations are an important field to study such rare disorders. While some mutations can lead to a varied range of diseases, they may also lead to variation and genetic recombination among generations. The study of such mutations helps in the precise pathogenesis and thereby efficient diagnosis and therapy. The diagnosis of several genetic disorders remains challenging, particularly in resource-limited areas. CRISPR/Cas systems can accurately identify and cleave particular DNA and RNA sequences, they have been utilized to modify genomes. Furthermore, some CRISPR/Cas systems show collateral nonspecific catalytic activity after recognizing the target sequence, which can be used for nucleic acid detection. Hence the genetic alterations are of great significance and allow accurate, highly sensitive, and in-field deployable diagnosis [5]. Further significant advances in CRIPSR technology is seen in the activity of Cas 13a for its collateral cleavage property. Hence CRISPR biosensing technology provides a promising future potential in diagnostics and efficient cancer mutation detection, genotyping and pathogen detection [6]. Several genetic disorders are also being treated using CRISPR-Cas systems, such as, the therapeutic properties of Cas9 improves the accuracy of gene editing and helps in reversal of gene mutation [7]. The application of this CRISPR/Cas systems have not yet been applied to the study of histiocytic gene disorders. Although several other effective therapies have been discovered for histiocytic and non-histiocytic neoplasms. For example, global paediatric health conducted certain tests on patients suffering from LCH. All 16 cases of initial symptoms were caused by bone lesions. Out of 16 patients, 8 of them showed soft tissue swelling while the other 8 had bone pain without swelling. All the vertebral body lesions and long bone lesions were accompanied by pain, while 4 of 6 cases of skull lesions were painless [8]. The histopathological tests of histiocytosis often show the presence of Cluster of differentiation 68( +) cells (CD68 is a protein expressed by cells in monocyte lineage, macrophages, and osteoclasts) along with varying amounts of tissue infiltration. Analysis of various samples showed *BRAF* (proto-oncogene B-Raf and v-RAF murine sarcoma viral oncogene homolog B) mutation as the primary oncogenic driver along with mutations activating the mitogen-activated protein kinase (MAPK) pathway. The discovery of the important role of the MAPK pathway has opened new research directions and therapeutic strategies [3]. A classification system for histiocytic disorders that effectively represents both the cellular origin and clinical characteristics (Fig. 2).

**Fig. 3:**
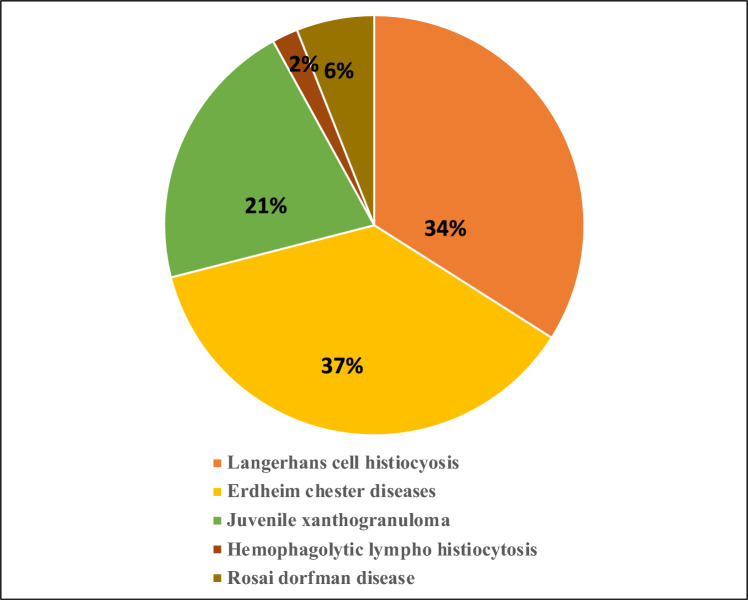
Prevalence of various histiocytic disorders (Recreated: [13])

In a 2022 study, it was discovered that MH had the highest occurrence of epigenetic gene mutations at 50%, compared to LCH, RDD, and ECD, which had 33%, 41%, and 45%, respectively [10]. The diagnosis age and organ involvement did not show any correlation with epigenetic gene mutations. The individuals with epigenetic mutations did not have a significantly different median overall survival (OS) from those without such mutations. This study encompassed a sizable sample of patients with various histiocytic and dendritic cell conditions, and it revealed a notable prevalence of epigenetic gene mutations at 41% [10, 11]. LCH is a rare and multifaceted disease that predominantly affects children. According to a study, 658 patients were identified with LCH, of whom 49% were children aged < 15 years. The age-standardized incidence rate was 4.46 per million children and 1.06 per million adults aged ≥ 15 years. The prevalence of LCH was 9.95 per million persons at the end of year 2019. The estimated annual incidence ranges from 0.5 to 5.4 cases per million persons per year, with approximately 1200 new cases per year reported in the United States. The frequency of LCH is greater in males than in females, with a male-to-female ratio of 2:1 [12], while, the prevalence of other non-langerhans cell histiocytosis is still under study, but the numbers for RDD, ECD, JXG, and HLH were reported to be 6%, 37%, 21%, and 2%, respectively, out of 270 patients studied [13] (Fig. 3).

**Fig. 4:**
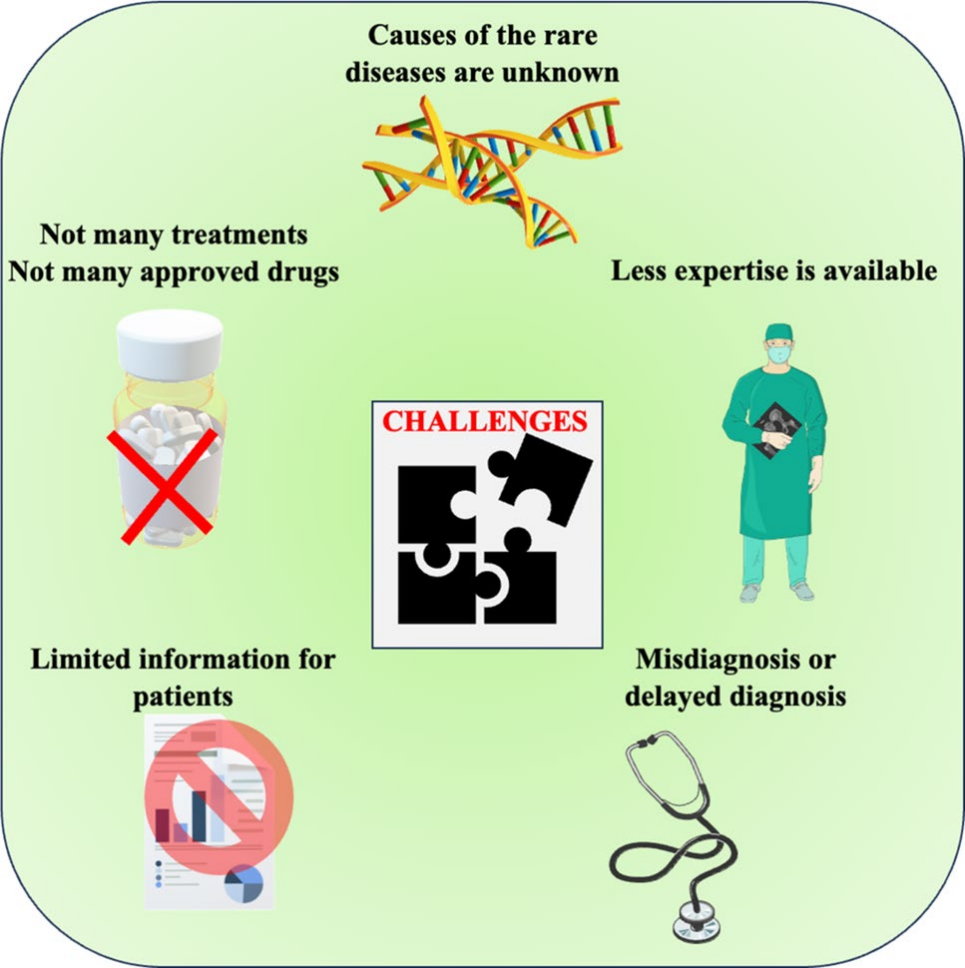
Challenges faced in diagnosis of histiocytosis. Parts of the figure were drawn by using pictures from Servier Medical Art. Servier Medical Art by Servier is licensed under a Creative Commons Attribution 3.0 Unported License (https://creativecommons.org/licenses/by/3.0/)

Rare disorders are not well-studied, which may lead to delayed or misdiagnoses (Fig. 4). These are often present with atypical symptoms, and healthcare professionals may lack the necessary experience and knowledge to recognize and diagnose these conditions promptly. This lack of awareness also extends to the affected individuals, making it harder to access appropriate medical care and support services.

Recent review articles majorly covered the LCH mutation and pathways. [1] article focuses about the histiocytosis, while also explained the major pathways such as MAPK pathway of LCH. Authors mentioned the lack of understanding of the other pathways in which mutations arise. Other mutations such as ALK, CSFIR, RET also give rise to lesions in histiocytosis. Few research articles also mentioned in brief about mutations in RDD, JXG and ECD [1, 14, 15]. Diamond et al. focuses mainly on the mutations of the various types of histiocytosis, while not going into detail about the treatment, symptoms and challenges faced [16]. Allen and Parsons discussed about LCH, JXG, and ECD, their mutations as well as their clinical symptoms. But the article does not elaborate on treatment of different types of histiocytosis [17].

Limited research work on genetic alterations in histiocytosis necessitates a thorough exploration of molecular mutations to achieve a better understanding. However, among the various other types of histiocytosis, considerable amount of work is done on LCH, one of the prominent types of histiocytosis, such as, the roles and implications of BRAF mutations in LCH pathogenesis. The research data illustrates how distinct these mutations provide novel prognostic insights and exploring their potential impact on tailoring personalized treatment strategies. Furthermore, these data act as good evidence for the discovery of targeted therapies based on identified molecular pathways. In conclusion, our review article summarizes the groundbreaking findings with respect to molecular mutations in histiocytosis and may act as a motivation for further pioneering research in the field.

Current review focuses on the genetic modification of LCH, RDD, ECD, HLH, and JXG in the patient samples that has been reported. It also discusses the diagnostic implications and molecular markers for histiocytosis diagnosis, as well as the advances and challenges in molecular diagnostics. Additionally, the prognostic insights and impact of genetic alterations on clinical outcomes are explored, along with the potential therapeutic implications of targeting molecular pathways for histiocytosis treatment. The study highlights the significance of understanding molecular mutations in histiocytosis research and identifies potential areas for further investigation and summarizes key findings.

## Langerhans Cell Histiocytosis (LCH)

The most prevalent form of histiocytosis is LCH. The inflammatory lesions pertaining to this disease are classified by the abundance of histiocytes which cause damage to the affected tissue [18]. It can be seen at any age from newborns to the elderly. The clinical involvement of LCH involves a wide spectrum of locations within the body, the most common one being bony lesions. Other locations include the skin, some soft tissue, lymph nodes, and thymus. Patients who show symptoms such as soft tissue swelling, external ear drainage, and increase in the size of the lymph nodes and thymus, and enlargement in the gum with early eruption of baby teeth can all be treated relatively easily. More serious symptoms involve the dysfunction of the liver, intestinal, and central nervous system involvement. The disease can vary from a single-system disease (affecting only a single organ) or a multisystem disease (affecting multiple organs). LCH is more commonly seen in children, however several cases are also seen in adults. Infants showing these symptoms are relatively hard to treat [19]. LCH in children is diagnosed with a combination of clinical, histological, and radiological conclusions. It involves both directly observed symptoms and results obtained from lab tests. Thus, it should be performed in a suitable setting to avoid any misdiagnosis [20]. Other tests to be performed include blood tests and imaging alongside choice tests based on the age and symptoms observed. The histology of the tissue is obtained from the site of disease through biopsy or surgical excision [21]. Common symptoms of the disease, such as skin lesions, and palpation of the lymph nodes, spleen, and liver are diagnosed in a clinical examination. These findings aim to establish the range of the disease. The infiltration of CD1a + , and CD207 + histiocytes is what classifies histology. The disease is then classified as either single (having affected only one organ) or multisystem (having affected many organs). Commonly a medical examination using magnetic resonance imaging (MRI) is done for children diagnosed with diabetes insipidus or for children showing any neurological symptoms. A computerized tomography scan is crucial in diagnosing pulmonary LCH. This shows the cavitated nodules and the prognosis of these nodules into cysts. Central nervous system magnetic resonance imaging (CNS-MRI) is suggested for all patients [1]. The prognosis of the disease depends on the organs affected and the response of the patient to the therapy offered [22]. Adult patients having a single system showed bone involvement, particularly in the ribs. It is associated with multisystem LCH in adults however does not show much correlation to single-system LCH [23]. The prognosis of single-system bone LCH is benign. Most patients with single-system LCH show good prognosis and respond to treatments well [24]. Single-system treatments including biopsy or surgical excisions of bone lesions are the most common local treatments for bone LCH and for pulmonary LCH, which affects the lungs, and is commonly linked to smoking, making smoking discontinuation a primary front-line treatment method. Multiple system therapy including radiotherapy and photochemotherapy, can be used in emergency situations such as symptoms of spinal cord compression and optic nerve compression [11]. Various types of medications, including steroids, non-steroidal anti-inflammatory drugs (NSAIDs), and cytostatic drugs, have been identified and employed in the treatment of LCH. Examples of these drugs include- vincristine, vinblastine, VP-16 (etoposide), 6-mercaptopurine (6-MP), methotrexate, cytarabine, and cladribine. Some of these drugs are also commonly used as front-line therapy [25, 26]. Therapies that are still being reviewed but have shown potential for treatment are known as experimental therapy. Experimental therapy can be a randomized collection of potentially compatible treatments or the use of new experimental drugs and methods. Due to research made in the past decade, it has been discovered that the *BRAF* inhibitor, vemurafenib, has shown positive results in certain cases, with the reduction of discomfort and symptoms [27].

**Fig. 5:**
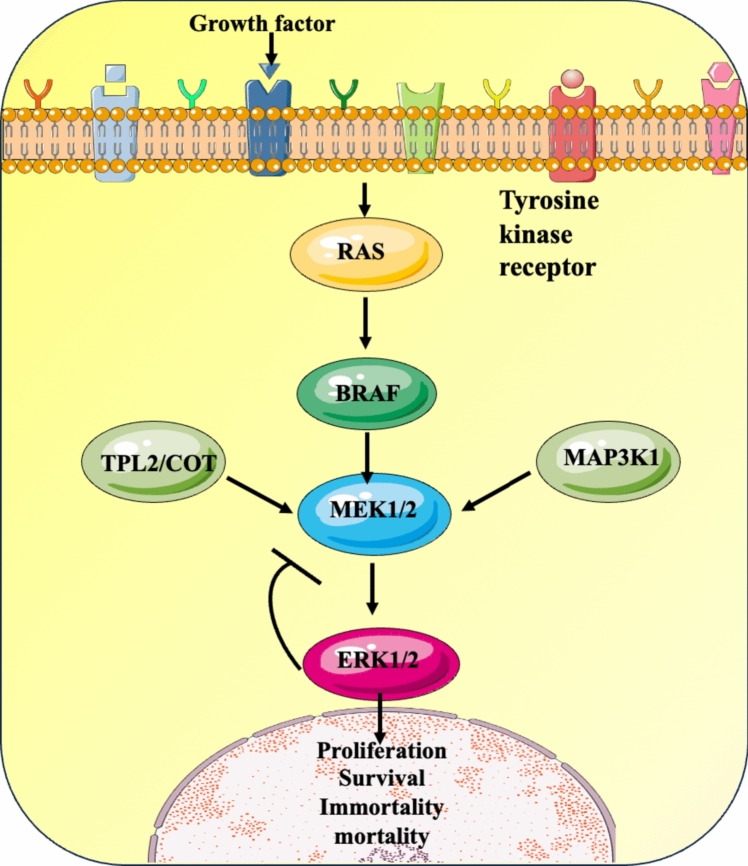
Frequently effected genes and pathways associated with LCH [35]

The most common MAPK pathway alteration in adults is the BRAF deletion [28, 29]. Oncogenic mutations discovered in the MAPK signalling pathway have transformed clinical therapy [1]. Approximately 55% of the LCH cases were known to have the *BRAF* ^V600E^ [proto-oncogene B-Raf and v-Raf murine sarcoma viral oncogene homolog B where valine (V) is replaced by glutamic acid (E) in the 600th position] mutation which activated the MAPK signalling pathway. Hence it was settled that the *BRAF* ^V600E^ is a driver mutation in LCH. In a study performed by Heritier (2017), whole-exome sequencing was used to look into nine LCH cases of individuals with no *BRAF* and *MAP2K1* (Mitogen activated protein kinase 1) mutation. This resulted in the discovery of a new somatic BRAF splicing mutation in two cases, out of which both were childhood LCH single systems, and the bone lesions had self-healing properties. The new somatic mutation showed a nine-base pair duplication which translated into a mutated protein with the insertion of three amino acids [18]. In another study performed by Badalian et al. [30], oncogenic BRAF V600E mutation was observed in formalin-fixed paraffin immersed material in 35 of 61 specimens. This was seen in samples of younger patients. Apart from the BRAF V600E, other mutations include MET E168D (Mesenchymal epithelial transition gene where glutamic acid (E) is replaced by aspartic acid (D) in the 168th position and TP53 R175H (Tumour protein coding gene where arginine (R) is replaced by histidine (H) in the 175th position [30]. The mutationally activated *BRAF* stimulates signaling through the rat sarcoma gene (RAS)/rapidly accelerated fibrosarcoma (RAF)/mitogen activated protein kinase pathway (MEK)/Extracellular signal-regulated Kinase (ERK) pathway resulting in constitutive gene transcription involved in a vast variety of cellular responses such as proliferation [31]. Hence RAS/RAF/MEK/ERK pathway inhibitors can be used to treat patients. In cases where *BRAF* mutation was absent, the ERK was still reported to be activated. The other mutations that activated the MAPK cell signaling pathway are the *MAP2K1*, β3-αC loop omission in the kinase domain of BRAF. Some cases have stated mutations on *ARAF* (Serine/threonine-protein kinase A-Raf) and *MAP3K1* [18]. MAP2K1 mutations include C121S and C121S/G128D in the kinase domain and 5661QKQKVG > R (N-terminal regulatory domain deletion in frame). These variant proteins lead to the phosphorylation of ERK in vitro kinase assays [32]. Genotyping LCH lesions using whole-exome sequencing (WES) or targeted gene panel next-generation sequencing showed that MAPK pathway alterations were seen in almost all the cases particularly in pulmonary langerhans cell histiocytosis (PLCH) patients [33]. PLCH is a type of lung disease seen in young adult smokers. Most areas of the lung are affected as a result of this disease which leads to a wide variety of phenotypes. Upon analysis of the PLCH tissues mutations of specific mitogen-activated protein kinases were identified. Mutations seen in patients with PLCH include MAP2K1 along with activating NRAS (Neuroblastoma RAS viral oncogene homolog) mutations. The NRAS mutations occurred alongside the BRAF V600E mutation [34] (Fig. 5).

## Rosai-Dorfman Disease (RDD)

It was first described as a benign condition involving the lymph nodes, discovered by Destombes as ‘sinus histiocytosis with massive lymphadenopathy’. Lymphadenopathy refers to a condition that causes the lymph nodes to grow or behave abnormally. RDD was earlier categorized under non-LCH. Now RDD is categorized as part of the “R” group of histiocytic disorders in the Histiocyte Society's 2016 revision of its classification. This comprises both sporadic and familial RDD (lesions on extracutaneous tissues). There are further four classifications of sporadic RDD: nodal, extranodal, RDD linked to immunological disorders or neoplasia, and unclassified types. Whereas RDD that only affects the skin belongs to the “C” category (lesions on cutaneous and subcutaneous tissues). Each of these disorders could be either multifocal or unifocal. Some theories say that RDD might be an immune syndrome that is controlled by various gene mutations or other immune system-related factors. The histopathological features, under light microscopy, help us observe the RDD macrophages showing characteristics like oval nuclei, a central distinct nucleolus, and a pale eosinophilic cytoplasm in abundance, along with emperipolesis (i.e., the engulfment of inflammatory cells that are still intact). Positive markers of RDD include S100, cyclin D1, and OCT2, while the staining appears negative for markers like BRAF V600E, CD1a, and langerin. The biopsy specimens of RDD often have a reactive inflammatory background that comprises numerous plasma cells, lymphoid aggregates, and fibrosis [36]. RDD was earlier considered a non-neoplastic histio-lymphoproliferative disease of the cervical lymph nodes. The involvement of extranodal sites was later recognized. Some common extranodal sites of disease comprise the skin, respiratory pathways, endocrine glands, gastrointestinal tract, bone, and breast. RDD presents several symptoms like fever, ill health, and weight loss along with hyperglobulinemia and an increased serum erythrocyte sedimentation rate (ESR). Immunological abnormalities are observed too in certain patients. Emperipolesis is another distinct characteristic of the RDD-affected tissues. It is a condition of having an intact cell inside another cell; the histiocytes contain intact lymphocytes or erythrocytes in their cytoplasm. It is considered the histologic hallmark of RDD. The central nervous system, skin, and lymph nodes are the most often affected organs [37, 38]. Its diagnosis is based on the combined results of immunophenotypic and morphologic findings, imaging, clinical presentations, and molecular alterations. Diagnosis is often done by collecting and examining the immunohistochemical samples of the affected tissues [39]. RDD is distinguished by a mass of activated histiocytes within the damaged tissues, depicting a variable frequency of emperipolesis. Successful prognosis can be based on the number of systems involved or affected, but RDD is frequently self-limited. It is observed that a small number of patients experience unfavorable prognoses because of locally aggressive lesions, dissemination, and refractory disease [40].

**Fig. 6:**
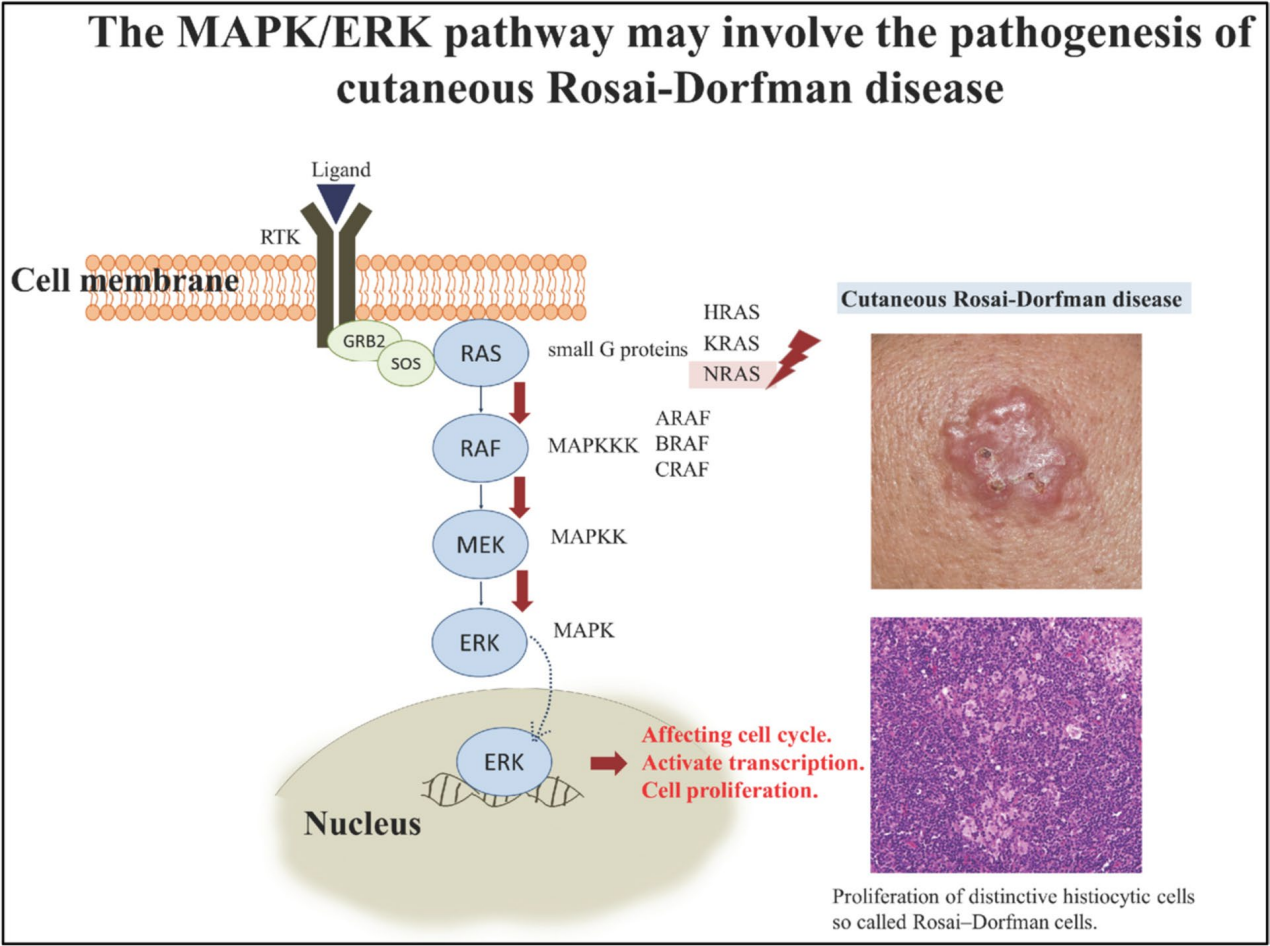
Pathway associated with cutaneous RDD (Adapted from [42])

RDD always accompanies an immune dysfunction such as RAS-associated autoimmune leuko-proliferative disease, idiopathic juvenile arthritis, systemic lupus erythematosus, and autoimmune haemolytic anaemia [41]. Cutaneous RDD is a proliferative disorder that is rare and is limited to the skin. Its pathogenesis remains unknown [42] (Fig. 6).

Nodal and extranodal sites of disease can be differentiated based on their magnitude of emperipolesis and fibrosis expression [43]. Anatomically delicate parts of the head and neck are most frequently involved in extranodal RDD involvement. Purely extra nodal RDD is always accompanied by a lymph node. The investigation of extremely few cases, especially those that are very contentious, is the cause of the poor etiology of RDD. RDD has always been regarded as reactive and non-clonal. However, RDD lesions have been documented to develop into high-grade lymphomas and histiocytic sarcomas, indicating the possibility of genetic instability [44]. No treatment has yet been approved by the United States Food and Drug Administration (US FDA) for RDD, so the patients are treated with a variety of relevant therapeutic agents [45]. Therapies for RDD may include corticosteroids as a front-line therapy, cladribine, 6-mercaptopurine, methotrexate, and azathioprine, in combination with prednisone are subsequent therapies that can be used. Rarely, pegylated interferon-Alphas are also used as a form of treatment [46, 47].

In recent years RDD has been recategorized as a neoplastic disease, one-third of patients have been identified with mutually exclusive Kirsten Rat Sarcoma viral oncogene homolog (*KRAS*) and *MAP2K1* mutations [45]. Lesioned tissues of RDD have shown *NRAS, KRAS, MAP2K1*, and *ARAF* alterations suggesting a clonal origin to the disease. Lesioned tissue should be examined to look for gain-of-function mutations in the MAPK pathway genes (at least *KRAS, NRAS, HRAS, ARAF, BRAF, and MAP2K1)* that are treatable with targeted therapy. Researchers have examined the mutations in these immunologic diseases (i.e., alteration in *FAS* (FAS cell surface death receptor*)* genes) and tried to relate them with the causes of RDD. Such cases (about 10%) have a male predominance and an onset at an early age [41]. The NGS analysis of patients by [36] depicted certain pathogenic mutations; Mutated *KRAS* p.G12D showed a gain of function (GOF) and a variant allele frequency (VAF) of 8.9%; Another patient had a cell division cycle (CDC) *73* truncation at exon 5 leading to loss of function (LOF) and a VAF of 3.6%; cases of *KRAS* p.K117N *and KRAS* p.A146T was observed inducing a GOF. Disorders like RDD and LCH that are associated with mutations of the MAPK pathway show overexpression of cyclin D1, a marker of neoplasia. The activity of cyclin D1 is correlated with the activity of sustained ERK (extracellular signal-regulated kinase). Although this is a useful diagnostic trait, it neither exclusively excludes reactive histiocytic neoplasms nor is it specific for RDD among histiocytic neoplasms. Recent evidence shows that RDD patients with MAPK pathway mutation can be effectively treated with MEK inhibitors [36]. Among all the patients under study by [45], five underwent next-generation sequencing (NGS). A case of *KRAS c.351A* > *T (K117N*) mutation was seen. Another showed a *CDC73* truncation in exon 5. It is interesting to note that two out of three patients with RDD/ECD overlap had a *MEK1* mutation [*MAP2K1 c.157 T* > *C(F53L)] [MAP2K1 c.167A* > *C (Q56P)*]. Unlike ECD and LCH, BRAF V600E mutations were found negative in all the cases [45]. A patient under study by Kala 2022 showed mutated *MAP2K1 and *Erb-B2 Receptor Tyrosine Kinase 2 (*ERBB2*) genes, suggesting *MAPK/ERK* pathway mutations as the most common alterations in RDD [38]. Rosemarie 2019 did a case study of a patient with mixed histiocytosis showing an overlap between RDD and LCH. One healthy 6 year old child complained about several months of neck pain and cervical lymphadenopathy. A biopsy was performed, and it was diagnosed with both LCH and RDD. Due to the close overlap of the LCH and RDD-affected cells, they both showed a dual expression of BRAF VE1, suggesting a common precursor *BRAF-*V600E precursor which gets differentiated along an unknown pathway in the later stages. Histopathological samples show large RDD histiocytes with sinus expansion and emperipolesis, intermixed with small LCH histiocytes having nuclear groves and an eosinophilic cytoplasm. The *BRAF-*V600E *(VE1*) antibody stained both types of histiocytes strongly. Though the frequency of reports of RDD cases with MAPK pathway alteration has been increasing, the *BRAF-*V600E mutations are still negligible in all but one case, i.e., the cases of overlap between RDD and LCH/ECD. Such patients show positive BRAF VE1 immunostaining in samples. Such overlaps are called ‘Mixed Histiocytosis’ and have varied definitions. The lesions can occur either in the same patient at different times or simultaneously at the same location in separate microenvironments [39]. Konstantinou and Tournier [48] presented a case of cutaneous RDD that showed the involvement of both the head and neck region harboring the Rearranged during transfection (receptor tyrosine kinase) (RET) gene (responsible for providing instructions to produce a protein involved in cell signaling) and the MAP2K1 pathway mutations. The patient was diagnosed with RDD based on the following histopathological observations; emperipolesis was observed along with many histiocytes containing red blood cells and lymphocytes. IHC staining came positive for S100, CD68, and pERK markers and negative for CD1a. The NGS detected an activating mutation of MAP2K1 c308T > A, p. (I103N) and another mutation of the RET c2371T > A, p. (Y791N). This case involved a simultaneous recognition of two mutated genes. Therefore, histiocytic neoplasms are now identified by two new genomic drivers- RET and ALK (Anaplastic Lymphoma Kinase) fusion genes. A tyrosine kinase receptor is encoded by the exons of the RET proto-oncogene. When RET is activated or under normal circumstances, it interacts by having its intracellular domain Y1062 phosphorylated, activating the downstream MAPK/RAS/ERK pathway. The RET c2371T > A mutation, which was found in the patient, has a well-documented pathogenic role in lymphoid malignancies. RET mutations may be the subject of a novel RDD therapy strategy. In one-third of patients with RDD with head and neck involvement and a multifocal disease, mutually exclusive KRAS and MAP2K1 mutations have been found, but not with the response rates to standard treatment [48]. Few studies have shown that cases with familial RDD may also show a germline mutation in *SLC29A3* (Solute Carrier Family 29 Member 3). A rare hereditary condition showing RDD-like lesions has been categorized under familial RDD, namely H syndrome or the Faisalabad syndrome. It is caused due to alterations in the nucleoside transporter gene *SLC29A3* and certain germline mutations in the genes that code for FAS. However, such mutations are not observed in sporadic RDD forms. Similarly, Neil 2010 also writes that those patients diagnosed with the syndromic types of histiocytosis—familial RDD and Faisalabad histiocytosis have been identified with *SLC29A3* germline mutations. Histopathological study of such cases shows conspicuous sinus histiocytes with pale cytoplasmic staining, large nuclei, and distinct emperipolesis. Hence, a research study of *SLC29A3* pathways seen in sporadic RDD cases will be of importance [49]. A genomic study of 21 RDD cases by Sofia 2017 identified *KRAS, NRAS, and ARAF* mutations as the major role players. 7 of 21 patients (33%) had mutations, including *KRAS* (*n* = 4) and *MAP2K1* (*n* = 3), and all 7 cases had point mutations. Two of the *KRAS-*mutated instances had exon 2 mutations, while two had exon 4 mutations*. KRAS* mutation allelic frequency was 4.69% on average (range: 3.53–5.64%). Two of the *MAP2K1*-mutated patients had exon 3 mutations, while one instance had an exon 2 mutation. The range for the allelic frequency of the *MAP2K1* mutation was 4.49–76.2%. In any instance*, KRAS and MAP2K1* mutations did not coexist. In a *MAP2K1*-mutated instance, an additional new somatic CDH1 (Cadherin 1) mutation with an allelic frequency of 8.75% was found. Levels of expression of pERK antibodies were studied by immunohistochemical analysis. The nucleus and cytoplasm of histiocytes in the ones affected by *MAP2K1* mutations showed high levels of expression. While cases with *KRAS* mutations had negligible expression. In every instance, stromal cells, fibroblasts, and endothelial cells were p-ERK-positive. Certain important cellular processes such as cell proliferation, survival, apoptosis, and cell differentiation targeted for the cell membrane, nucleus, or cytoplasm, are regulated by the MAPK/ERK pathways. In a simplified model, the G protein RAS on activation triggers the phosphorylation of the serine/threonine kinases ERK and the downstream kinases RAF and MEK. This phosphorylated ERK (activated) transports to the nucleus and actively regulates the functioning of some transcription factors, which are responsible for the expression of genes for proliferation and survival. It can hence be established that any alterations in this pathway can become a cause of non-hematologic malignancies such as carcinomas, melanoma and even histiocytosis (ECD, LCH, RDD) [40]. Shanmugam et al. [44] reported a case of RDD that was due to a KRAS K117N missense mutation that involved the submandibular salivary glands. The patient was a Canadian man, who was 45 years old. A biopsy of his affected gland showed emperipolesis and round vesicular nuclei with prominent nucleoli and histiocytic cells with foamy cytoplasm, thereby aiding in the diagnosis of RDD. Imaging also showed lymphadenopathy. The NGS depicted KRAS mutation with a variant allelic frequency of 11% [44]. The clinical study of KRAS is of great importance with respect to cancers, somatic GOF mutations in this gene are often observed in about 25% of cancers affecting humans*.* KRAS*,* a regulatory Guanosine triphosphatase (GTPase), plays an essential role in cell cycle control and signal transduction. KRAS gets activated once bound to Guanosine triphosphate (GTP) and in turn, activates downstream signalling factors. GTP hydrolyzes to Guanosine diphosphate (GDP) leading to KRAS inactivation. Gain-of-function *KRAS* alterations cause the loss of ability of GTP to hydrolyze, leading to continuous KRAS-GTP association and persistent activation of downstream transcription factors [50]. Wu et al. [42] shown specifically in cases of purely cutaneous RDD, which was then concluded to be a separate entity due to its distinctness from the typical RDD in histopathology, epidemiology, and symptoms. Cases with purely cutaneous RDD show less emperipolesis along with brown discoloration and hyperpigmentation observed in the immunohistochemical samples, greater extent of stromal fibrosis and more persistent infiltration of plasma cells. Mutations in these cases were found in the Kirsten Rat Sarcoma 2 viral oncogene homolog (*KRAS*), and *NRAS* in the MAPK pathway. For this study, 7 patients with purely cutaneous RDD were studied. 6 out of the 7 cases detected positive for NRAS alterations such as the *NRAS* A146T (*n* = 4/7) and *NRAS* G13S (*n* = 2/7). While no *BRAF or KRAS* mutation was detected. This concluded that *NRAS* alteration is the most prominent point mutant in cutaneous RDD patients. Certain distinct mutations are found in the mitogen-activated protein kinase/extracellular signal-regulated kinase (MAPK/ERK) pathway including the *BRAF V600E*, *KRAS*, *MAP2K1* and lastly *NRAS*. To sum up, the major mutations playing a role in the parthenogenesis of RDD involve *NRAS, MAP2K1*, and *ARAF.* A previous study documented a case wherein a patient with autoimmune lymphoproliferative syndrome developed histiocytic sarcoma in the presence of RDD, accompanied by a germline missense mutation in exon 9 of the Tumour Necrosis Factor Receptor Super Family 6 (TNFRSF6) gene responsible for encoding *FAS*. This finding shed light on likely pathogenesis involving the *FAS* ligand pathway [42]. Another study stated that out of the 37 patients investigated for non-Langerhans histiocytosis, 8 patients were diagnosed with RDD out of which 50% of cases harbored mutually exclusive mutations of *ARAF, NRAS, or KRAS*. After several research studies, it was assumed that activating alterations in the RAF/RAS/ERK/MAPK or any other similar signaling pathway may be the key player in the pathogenesis of RDD, concluding that it is a histiocytic disorder activated by genetic mutations [40]. A rise in RDD cases has been observed over the years. Therefore, further research into the mutational profile of RDD and its therapeutic implications is urgently needed. Due to the unclear pathogenesis of RDD a debate still goes on, on whether it is a benign or a neoplastic disorder. The MAP-ERK pathway mutations found in about one-third of patients indicate that at least a subgroup may be neoplastic in nature. With a tendency of RDD to form masses, few researchers consider it neoplastic. It is believed that RDD will resolve on its own and does not require urgent treatment. However, some individuals may have stubborn or recurring symptoms. Treatment options for skin-related (cutaneous) RDD include systemic corticosteroids, methotrexate, radiotherapy, surgery, intralesional or topical corticosteroids, acitretin, or, cryotherapy. RDD appears to have a low fatality rate, but if left untreated, it has the potential to be fatal due to end organ damage and considerable morbidity. This disease has now been known for five decades; however, its etiology still remains poorly understood. The importance of the MAPK/ERK pathway in RDD can be highlighted by the impact and use of MEK inhibitors in the treatment of patients. Jacobsen et al. stated an RDD case with *KRAS* mutation that was treated with cobimetinib, an MEK inhibitor [42]. MEK inhibition is the most widely used treatment for adults with *KRAS*-mutated RDD. The application and potential of targeted treatments and tumor sequencing for RDD have not yet been explored. Additionally, one *MAP2K1*-mutated instance was found to have a unique somatic mutation in the cadherin 1 gene (*CDH11* L71F); however, the biological and clinical relevance of this result remains unknown. Garces et al., have discussed a possible connection between RDD and IgG4-related disorders accompanied by certain autoimmune disorders [40]. Recent studies have shown rare cases of involvement of the central nervous system (CNS). Reasons as to why it shows a strong male predominance are under study. Improvement in the specificity of magnetic resonance spectroscopy may have a future prospect for pre-operative diagnosis in patients affected with RDD [43].

## Erdheim-Chester Disease (ECD)

ECD is an extremely uncommon form of histiocytosis that does not involve the Langerhans cells. The main defining factor of ECD is the infiltration of tissues by foamy histiocytes. The initial diagnosis for ECD for this branch of histiocytosis involves a thorough physical examination supported with lab tests, additional diagnosis of ECD involves the biopsy of tissue and analysis of the histiocytosis present in them, the histiocytes are typically foamy (histiocytes contain wastes from other cells present in them) and contain CD68 + and CD1a − which set them apart from other types of histiocytosis [51, 52]. Adults suspected to have ECD or with confirmed ECD are suggested a positron emission tomography -CT scanning. CT scanning helps us detect lung, vascular, retroperitoneal infiltration, and interlobular septal thickening [53]. It also reveals retroperitoneal fibrosis, which is a characteristic symptom of ECD, it is also referred to as hairy kidney. An MRI of the heart and brain is also suggested which helps diagnose the unseen physical symptoms such as pleural, pericardial, or peritoneal thickening. Steroids, cytotoxic drugs, and autologous hematopoietic stem cell transplantation are the front-line treatments for ECD, however, they have shown very limited clinical efficiency*. BRAF* inhibitor- vemurafenib has shown potential as a source of treatment. Interferon-alpha is also another treatment option viable for ECD [54].

A *BRAFV600E* gene mutation was found in most cases of ECD, which has provided a lot of insight into the disease itself [46]. *BRAF* mutations are present in several non-melanoma cancers, including colorectal cancers, non-small-cell lung cancers, hairy-cell leukemia, multiple myeloma as well as LCH [55, 56]. Clinical symptoms of ECD include bone pain, diabetes insipidus as well as neurological and constitutional symptoms. There have been cases of cutaneous, cardiovascular, and pulmonary involvement, although most of the sites of occurrences are retroperitoneal [51]. The diagnosis of ECD is a difficult task due to the lack of sufficient knowledge about the disease as well as the rare nature of the disease itself. However, there are a few methods that can be used to diagnose ECD, biopsy being the most efficient method for the identification of ECD. Another key characteristic/defining factor of ECD is the thickening of spongy bone (osteosclerosis) across the metaphyseal and diaphyseal regions of the long bones present in the lower regions of the body [57, 58]. Some cases of ECD have a neurological symptom component, as when a lesion appears on the brain due to the infiltration of histiocytes a few symptoms include speech impediment, incoordination between limbs, and gait ataxia. Impairment of balance is also a common symptom in patients of ECD [59]. There have also been cases of urological symptoms involved in cases of ECD. It was also further discovered that some of the patients suffering from ECD had an involuntary activation of the mitogen-activated protein kinase (MAPK) series of pathways that play a role in regulating a wide variety of cellular responses, which include apoptosis, proliferation, differentiation, and a variety of stress responses in cells [56]. There are 3 main types of kinases present in the MAPK family, the first is the standard MAPK, then MAPK Kinase (*MAP2K*), and finally, MAPK kinase kinase (*MAP3K*), which all play an important role in the activation of downstream proteins and their phosphorylation and ECD [60, 61]. The *BRAF V600E* mutation where the glutamic acid at the 600th position of the protein BRAF, is substituted with a valine group is heavily linked to ECD as well BRAF being a protein that is involved in the RAS/RAF/MEK/ERK pathways [62, 63]. There have been some cases of ECD without the *BRAF* mutation, however, there also has been a relation between ECD and acute myeloid leukemia due to their similar origin arising from mutations in genes. As mentioned above in the treatments, the BRAF inhibitor- vemurafenib has been used to slow the pace of ECD which also provides further evidence between the mutated gene and the disease. In addition to the *BRAF V600E* gene mutation, a large number of recurrent mutations have been found in ECD patients, these include *MAP2K1, ARAF, NRAS, and KRAS* mutations which have been found to occur in at least 30% of patients with ECD [17, 55, 64]. MAP2K1 generally codes for the dual-specificity kinase MEK1 protein, which is then activated by BRAF in the MAPK pathway. However, it is extremely important to note that although BRAF and MAP2K1 mutations are mutually exclusive, they both serve as an initiator for ECD/LCH to move down the same pathway [46]. *MAP2K1* mutations can arise from a variety of sources from the deletion of a sequence of base pairs in the DNA. A majority of them are normally in-frame deletions [65]. Another gene with a high mutation rate is KRAS, found on chromosome 12 (12p11.1-12p12.1). KRAS mutations are predominantly single-base missense mutations which are commonly found on codon 12 (G12), codon 13 (G13), or codon 61 (Q61). However, there are also many subtypes of *KRAS* mutations, such as substitution mutations. The most common substitution is glycine to cysteine (G12C) [66]. Phosphatidylinositol 3-kinase (*PIK3CA*) gene mutations have also been identified in studies performed by Emile et al. however the occurrence of the gene mutation is not as high as that of the *BRAF* V600E mutations, which were found present in 47.5% of 80 cases, compared to *PIK3CA* gene mutation was found in only 7 of the 58 ECD patients, of which 4 are *BRAF* mutated. Understanding the relation between the *PIK3CA* mutations and clinical treatments might provide a breakthrough regarding the treatment of ECD [67]. *NRAS and ARAF* mutations have been found in an extremely small number of cases, a study was performed by Micol et al. on 37 cases of *BRAF* V600E wild-type mutations. Only 1 of these cases reported an *ARAF* mutation while 6 cases reported a *NRAS* mutation. The mutation of these genes is yet to be studied [16]. An additional rare type of gene mutations present are gene fusion mutations, although not much information is available, several of them exist and they alter the function of pathways differently than regular gene mutations. *BRAF* fusion mutations can act as an alternate method of ERK pathway activation. *BRAF* fusions include Protein kinase C and casein kinase substrate in neurons protein 2 (*PACSIN2*)–*BRAF* fusion which affects the bone and skin, Bicaudal D cargo adaptor (BICD2)-BRAF fusion which affects the brain and Ubiquitin Domain Containing 2 (*UBTD2*)*-BRAF* fusion affects the brain and bone. Several BRAF fusion mutations have been described by [68]. ALK-positive histiocytosis is an extremely recent discovery with the first case being reported 14 years ago. ALK fusions are even rarer, with targeted NGS techniques being used to identify Kinesin Family Member 5B (*KIF5B*)*-ALK* fusions in a small number of cases. Other fusions involved are *BRAF, MAP2K1, KRAS, NRAS, and PIK3CA*. *ALK* gene fusions have also been discovered in a subset of other histiocytic neoplasms as well as in a variety of other human tumors [69]. While we have a very small understanding of ECD, prospects of understanding the molecular features of ECD and other diseases that could be related to ECD (e.g., hairy-cell leukemia, malignant melanoma). We can effectively attempt to use treatments for the related diseases in ECD patients. This has been done with varying levels of success, but a cause-and-effect relationship has been proven between the various diseases and relationships, proving to us that the nature of the diseases is linked to each other [70]. The extent of the linkage is also another future prospect that could be a possible area of research exploration. Another field of research, regarding ECD, is the effect of various pathway inhibitors in the development of the disease [71].

## Hemophagocytic Lymphohistiocytosis (HLH)

Perforin gene (PRF1) mutations have been seen in some HLH patients, being the most common mutation found. A variety of mutations in the PRF1 gene can cause HLH, one case specifically involved deletions of two codons at 1090 and 1091 and a conversion of bases at position 916 from guanine to adenine. PRF1 mutations significantly affect the expression of perforins in the cells, leading to a decrease in the number of perforins in patients with HLH arising from several PRF1 mutations. Methods for analysis of PRF1 gene include flow cytometry, assay for cell Natural Killers Cells (NK) activity, and sequencing of PRF1 [72]. Treatment of HLH is also varied, mostly depending on the severity of the symptoms and the underlying cause of the disease. The earlier treatments of HLH used to involve immune-suppressive agents and modulatory agents, and if the HLH is caused by the onset of an illness such as in acquired HLH, treatment of the causative agent/disease. The methodology of the treatment is to reduce the inflammatory responses caused by the cytokines and then a subsequent dysregulation of the immune system to reduce the extent of the damage caused by the disease or to destroy the antigen causing the induced HLH [73, 74].

Perforin forms channels on the surface of the target membrane, using Ca^+^ ions. Perforin is commonly found in CD16 + /CD56 + NK cells and hence any depression in the perforin in these cells might indicate the presence of HLH. Due to this, perforin expression in these cells has been used as a diagnostic criterion for HLH [75]. Studies have found that different gene mutations result in the formation of different types of F-HLH. *PRF1* mutations generally cause FHLH (Familial Hemophagocytic Lymphohistiocytosis) type 2, *UNC13D* (Protein unc-13 homolog D) mutations cause FHLH type 3, *STX11* mutations cause F-HLH type 4, *STXB2* (Cellular receptor of the B-subunit of Shiga toxin) mutations cause F-HLH type 5. In addition, linkage analysis of genetic defects with HLH has shown the locus for F-HLH type 1 being present on 9q21.3–22, however the causative gene is yet to be discovered, providing a possible future prospect on the advancement of HLH detection. These mutations generally cause a change in the transport, membrane fusion, or exocytosis capabilities of perforin/granzyme of lytic granules of natural killer cells and cytotoxic T Lymphocytes [76]. HLH is an extremely dangerous and possibly fatal group of disorders that are characterized by the constant, unregulated activation of cytotoxic T lymphocytes, NK, and macrophages. This causes an immune-mediated response that attacks the host cells leading to damage of multiple organ systems. An additional effect is the cause of a cytokine storm, which is the release of a large amount of cytokines (inflammatory cells), causing multisystem organ damage as well as cell death. It is caused by genetic mutations that lead to the improper functioning of perforins and granzymes, which both play an important role in the body’s responses to viral and tumor cells [77]. The Histiocytosis Society in 1994 had come up with a standardized set of diagnostic criteria for the identification of HLH, however, this was later revised in the 2004- edition for the diagnosis of HLH. These criteria include- hyperferritinemia (presence of high amounts of ferritin in the blood), Soluble interleukin-2 receptor is also an extremely good, inexpensive test for the confirmation of HLH, hyperbilirubinemia, hepatomegaly and elevated lactate dehydrogenase levels are also used in the diagnosis of HLH. Mutations in genes associated with HLH can also be used to identify HLH, the most common mutations being the *PRF1, UNC13D* and *STX11* gene mutations. Lymphoma can also act as a trigger for HLH, but are also hard to detect, various techniques can be used to bypass this and possibly screen for HLH. These techniques also include the use of positron emission tomography-guided imaging, repetitive tissue sampling and a consultation with a specialist in lymphomas [78]. HLH can also be classified into two types based on the causative agent of the disease- Primary and Secondary. Primary HLH is also known as Familial HLH (F-HLH), which is caused by genetic aberrations that are usually inherited in a heterozygous or homozygous manner. F-HLH has also been related to several immunodeficiency disorders. Secondary HLH is the result of an external stimulant such as infection, malignancy, rheumatologic disease, post-allogeneic hematopoietic stem cell transplantation, etc. Hence it is also called acquired HLH, the most common trigger for secondary HLH is the Epstein-Barr virus (EBV) [79].

## Juvenile Xanthogranuloma (JXG)

JXG is a very uncommon disorder, however, it is classified in the non-LCH group of disorders which is relatively large compared to the other groups of classification and JXG occupies a large number of cases within this group. It normally occurs within the first year of life but in certain cases, it may arise from the moment of birth itself. JXG is further divided into two groups- cutaneous juvenile xanthogranuloma (C-JXG) and systemic juvenile xanthogranuloma (S-JXG). C-JXG usually follows a benign course with symptoms listed above, it does not require treatment and is normally self-limited, the symptoms usually regress over a period of three years, although the timing is variable. S-JXG generally occurs without prominent cutaneous manifestations, however, in the rare cases that skin lesions are present, they generally precede the development of the other symptoms. S-JXG affects two or more organ systems in variable combinations and occurrences, in addition, some of these sites may be asymptomatic but will be affected and cause clinical problems [80, 81]. JXGs are usually mistaken as moles at birth, because it normally occurs as solitary lesions. The papules or nodules of JXG are usually well-demarcated, firm, rubbery, round to oval papules or nodules varying from 0.5 to 2.0 cm. During the earlier stages of the disease, it has a pink-reddish colour which will eventually develop a brown hue. In addition, JXG can be divided into 2 groups based on the size of the papules—small nodular (0.2–0.5 cm) and large nodular (1–2 cm) [82].

The main characterization of JXG is the presence of one or two nodules with predilection sites on the head and the neck, although various sites have also been reported with these two sites being the most common. The skin lesions, which are reddish or yellowish benign papules or nodules, are usually self-limited and do not require treatment. JXG is histologically composed of histiocytes, foamy cells, and touton giant cell collections. Diagnosis is most efficiently done via biopsy but can also be done clinically [83]. Novel somatic mutations of *MAPK1* have been identified in some cases of JXG with most of them being missense mutations in 74% of 89 cases. The frequency of *MAPK1* mutations appears to be higher than any other mutation except for the wild-type mutations. In a case sample of 11 JXG patients with 4 of them being paediatric, 27% of these cases had a *MAPK1* mutation [84]. The BRAF gene is known to be involved in most histiocytic disorders, JXG being no exception, in fact, some cases have the same exact *BRAF* V600E mutation that ECD and LCH follow. These cases have been described by Techavichit et al. and have the common characteristic of cranial/intracranial lesions across all 3 cases containing a *BRAF* V600E mutation. However, in regular cases of JXG with cutaneous symptoms, the V600E mutation was absent but a variation of *BRAF* mutation could be present [85].

*ALK* translocations are more frequent in S-JXG than the *BRAF* mutations, according to a sample taken by Xu et al. since *ALK* translocations and *BRAF* mutations are mutually exclusive, a study was performed and it was shown that among 12 patients with molecular alterations in JXG, the amount of ALK translocations was double than that of *BRAF* mutations with 8 cases of *ALK* translocations compared to the 4 cases of *BRAF* mutations. The reason behind this is not completely understood yet, but it could provide a future scope into the treatment of S-JXG [86]. Several gene mutations have been linked to JXG, including *MAP2K1, *Colony Stimulating factor 1 receptor (*CSF1R*)*, ALK, MET, CSF3R, KRAS, NRAS, KIT* (Tyrosine Kinase Receptor type III)*, JAK3* (Tyrosine protein kinase JAK3)*,* and a number of gene fusions. *CSF1R* mutations have been found to occur in approximately 10% of JXG cases, as demonstrated in research conducted by Durham et al. intracellular alterations in *CSF1R* leading to receptor activation have not been described [13]. *KRAS* mutations have been identified in patients with multifocal skin involvement, in studies conducted by Helias-Rodzewicz et al. two cases of the mutations have been observed in sample size of 21 patients. *KRAS* G12R *and KRAS* Q70 are the two mutations observed in the experiment. All the mutations present in JXG are extremely vague with little to no data regarding the processes of the gene, however, the presence of the genes is guaranteed due to the tests used for NGS, BRAF analysis, histological review, etc. [87]. Neurotrophic tropomyosin-receptor kinase (*NTRK*) gene mutations are also relatively common to JXG. All *NTRK*-positive cases of the study conducted by umphress et al., had a solitary lesion with no multifocal aspect. *NTRK* fusions are much less common to their lone counterpart, with two being described in the same study *TMP3-NTRK1* and *PRDX1*(Peroxiredoxin-1)*-NRTK1* being the two found, however there is a possibility that other fusions could arise from *NTRK* subtypes such as *NTRK3* [88]. Table 1 shows the mutations observed in the different types of histiocytosis.

**Table 1:** Various types of mutation seen in different histiocytic disorders

Type of histiocytic disorder	Total cases studied	No. of patients positive	Mutations	Reference
Langerhans cells histiocytosis (LCH)	30	20	*MAP2K1*	[32]
	9	2	*ARAF* and* MAP3K1*	[18]
	34	1	*MET* E168D	[30]
		1	*TP53* R175H	
		17	*BRAF* V600E	
	26	17	*NRAS, MAP2K1, BRAF*V600E	[34]
Rosai-Dorfman disease (RDD)	33	6	*KRAS p.K117N, KRAS p.G12D, KRAS p.146 T, CDC73* truncation of exon 5	[36]
	64	5	*KRAS c.351A* > *T, MAP2K1 c.157 T* > *C, MAP2K1 c.167 A* > *C*	[45]
	19	2	*MAP2K1, ERBB2*	[38]
	1	1	*BRAF* VE1	[39]
	1	1	*MAP2K1 c.308 T* > A, RET c2371T > A	[48]
	1	1	*KRAS* K117N missense mutation	44
	37	8	*KRAS, ARAF, NRAS, TNFRSF6*	[40]
	3 families	8	*SLC29A3*	[49]
	7	6	*NRAS* A146T*, NRAS* G13S	[42]
Erdheim-Chester Disease (ECD)	18	18	*BRAF* V600E	[62]
	21	10	*PIK3CA and NRAS*	[67]
	126	23	*BRAF* fusions	[68]
Hemophagocytic Lymphohistiocytosis (HLH)	1	1	*PRF1, UNC13D, STX*	[79]
Juvenile Xanthogranuloma (JXG)	11	3	*MAP2K1*	[84]
	11	2	*NRAS*	
	11	2	*KRAS*	

## Conclusions

Diagnosing histiocytosis necessitates a comprehensive approach that integrates clinical assessment, specialized examinations, and the collaboration of medical experts. Given the rarity and intricacy of these conditions, arriving at a diagnosis can be a formidable task, often demanding a deeper comprehension of epigenetic mutations and pathways involved. Among adults, the most prevalent alteration in the MAPK pathway is the deletion of *BRAF*. Around 55% of cases of LCH are known to exhibit the *BRAF V600E* mutation, which triggers the activation of the MAPK signaling pathway. Recent years have seen a reclassification of RDD as a neoplastic disorder. Out of 37 patients one-third of RDD patients had mutually exclusive *KRAS* and *MAP2K1* mutations [45]. The incidence of RDD cases has been on the rise, underscoring the urgent need for further research into the mutational profile of RDD and its therapeutic implications. The debate surrounding RDD's pathogenesis continues, with questions lingering about whether it should be classified as a benign or neoplastic disorder. In most of ECD cases, the presence of a *BRAF V600E* gene mutation has offered valuable insights into the disease itself. *BRAF* mutations are also observed in various non-melanoma cancers, including colorectal cancers, non-small-cell lung cancers, hairy-cell leukemia, multiple myeloma, and LCH.

Mutations in genes associated with hemophagocytic HLH can serve as markers for identifying HLH with *PRF1, UNC13D,* and *STX11* gene mutations being the most common. These mutations are linked to different types of familial HLH (F-HLH), with PRF1 mutations typically associated with F-HLH type 2, *UNC13D* mutations with F-HLH type 3, *STX11* mutations with F-HLH type 4, and *STXBP2* mutations with F-HLH type 5. JXG has been associated with several gene mutations, including *MAP2K1, CSF1R, ALK, MET, CSF3R, KRAS, NRAS, KIT, JAK3*, and various gene fusions. However, *CSF1R* mutations are found in approximately 10% of JXG cases. Intracellular alterations in *CSF1R* that lead to receptor activation have not been thoroughly described. Most of the mutation in histiocytosis is *BRAF V600E* in subtypes. The review provides valuable insights for enhancing therapeutic diagnostics. Histiocytosis identification has advanced significantly due to the identification of molecular mutations and genetic alterations. This knowledge has not only improved diagnostic accuracy but has also paved the way for more precise and effective treatment strategies. However, there is still much to learn about the genetic underpinnings of this diverse group of diseases, and further research is essential to unravel the complexities of histiocytosis and improve the lives of those affected.

Currently, the diagnosis and treatment of histiocytosis is extremely limited due to the lack of knowledge of the disease as well as the relatively underdeveloped technology. Moreover, understanding the molecular mechanisms that lead to the development of histiocytosis could provide a method of identifying early onset of histiocytosis. Therefore, advanced research on this field is essential. The study of histiocytosis and its mutations may help in understanding the gene interactions in cancer and may lead to the development of novel treatment methods for neoplasms.

## Data Availability

Not applicable.

## Abbreviations

LCH: Langerhans cell histiocytosis
CNS: Central nervous system
MH: Malignant histiocytosis
ECD: Erdheim chester disease
JXG: Juvenile xanthogranuloma
RDD: Rosai dorfman disease
HLH: Hemophagocytic lymphohistiocytosis
CD: Cluster of differentiation
BRAF: Proto-oncogene B-Raf and v-Raf murine sarcoma viral oncogene homolog B
MAPK: Mitogen activated protein kinase
OS: Overall survival
MRI: Magnetic resonance imaging
NSAID: Non-steroidal anti-inflammatory drug
VP-16: Etoposide
6-MP: 6-Mercaptopurine
MAP2K1: Mitogen activated protein kinase 1
BRAF^V600E^: Proto-oncogene B-Raf and v-Raf murine sarcoma viral oncogene homolog B where valine (V) is replaced by glutamic acid (E) in the 600th position
MET^E168D^: Mesenchymal epithelial transition gene where glutamic acid (E) is replaced by aspartic acid (D) in the 168th position
TP53^R175H^: Tumour protein coding gene where Arginine (R) is replaced by Histidine (H) in the 175th position
RAS: Rat sarcoma gene
RAF: Rapidly accelerated fibrosarcoma
MEK: Mitogen activated protein kinase pathway
ERK: Extracellular signal-regulated Kinase
WES: Whole exome sequencing
PLCH: Pulmonary langerhans cell histiocytosis
NRAS: Neuroblastoma RAS viral oncogene homolog
TPL2/COT: Tumour progression locus 2
ESR: Erythrocyte sedimentation rate
US FDA: United States Food and Drug Administration
KRAS: Kirsten rat sarcoma viral oncogene homolog
FAS: FAS cell surface death receptor
NGS: Next-generation sequencing
GOF: Gain of function
LOF: Loss of function
VAF: Variant allele frequency
CDC: Cell division cycle
ERBB2: Erb-B2 receptor tyrosine kinase 2
RET: Rearranged during transfection (receptor tyrosine kinase)
ALK: Anaplastic lymphoma kinase
SLC29A3: Solute carrier family 29 member 3
CDH1: Cadherin 1
GTP: Guanosine triphosphate
GDP: Guanosine diphosphate
NRAS: Neuroblastoma RAS viral oncogene homolog
TNFRSF6: Tumour necrosis factor receptor super family 6
ARAF: Serine/threonine-protein kinase A-Raf
BICD2: Bicaudal D cargo adaptor 2
PACSIN2: Protein kinase C and casein kinase substrate in neurons protein 2
PIK3CA: Phosphatidylinositol 3-kinase
UBTD2: Ubiquitin domain containing 2
KIF5B: Kinesin family member 5B
NK: Natural killer cells
PRF1: Perforin 1
UNC13D: Protein unc-13 homolog D
FHLH: Familial hemophagocytic lymphohistiocytosis
STXB: Cellular receptor of the B-subunit of Shiga toxin
EBV: Epstein barr virus
CJXG: Cutaneous juvenile xanthogranuloma
SJXG: Systemic juvenile xanthogranuloma
CSF1R: Colony stimulating factor 1 receptor
NTRK: Neurotrophic tropomyosin-receptor kinase
PRDX1: Peroxiredoxin-1
KIT: Tyrosine kinase receptor type III
JAK3: Tyrosine protein kinase JAK3
